# Family Satisfaction With Critical Care in The United Kingdom

**DOI:** 10.1186/2197-425X-3-S1-A22

**Published:** 2015-10-01

**Authors:** SE Wright, SE Harvey, E Walmsley, P Ferrando-Vivas, DA Harrison, KM Rowan

**Affiliations:** Freeman Hospital, Newcastle upon Tyne, United Kingdom; Intensive Care National Audit & Research Centre, Clinical Trials Unit, London, United Kingdom

## Introduction

A number of tools have been developed to seek the views of family members of critically ill patients but the most widely validated is the Family Satisfaction in the Intensive Care Unit 24-item questionnaire (FS-ICU-24),[1] which assesses overall family satisfaction, satisfaction with care and satisfaction with decision-making.

## Objectives

To assess family satisfaction with critical care in the United Kingdom using the FS-ICU-24, compare results internationally, and explore the impact of family, patient and other factors on comparisons between ICUs.

## Methods

The Family Reported Experiences Evaluation (FREE) Study recruited family members of patients staying at least 24 hours in 20 participating adult general ICUs between May 2013 and June 2014. Consenting family members were sent a postal questionnaire three weeks after the patient died or was discharged from ICU. Up to four family members were recruited per patient. Multilevel multivariable models were used to identify factors associated with satisfaction.

## Results

12,346 family members of 6380 patients were recruited to the FREE Study and 7173 (58%) family members of 4615 patients returned a completed questionnaire. Multiple imputation of missing item response enabled inclusion of all responders. Overall, satisfaction scores were high (mean overall family satisfaction 80, satisfaction with care 83, satisfaction with decision-making 75 out of 100) and were similar to other reports, internationally. Satisfaction was higher for family members of ICU non-survivors. Factors associated with overall satisfaction for family members of ICU survivors were family member age, ethnicity, relationship (to patient), visit frequency and patient acute severity of illness and receipt of invasive mechanical ventilation. Factors associated with overall satisfaction for family members of ICU non-survivors were patient age, acute severity of illness and duration of ICU stay. No other factors (of those explored) were associated. Significant variation existed across ICUs which reduced following adjustment for family and patient factors, resulting in fewer ICUs being identified as potential outliers.

## Conclusions

The large sample size and robust multilevel multivariable modelling of factors associated with overall satisfaction indicated the need for adjustment for these when comparing ICUs. The FREE Study and the FREE Study database are an important foundation and resource for future studies evaluating family satisfaction with critical care in the United Kingdom.

## Grant Acknowledgment

NIHR Health Services and Delivery Research Programme.
